# Prediction of Wave Transmission Characteristics of Low-Crested Structures with Comprehensive Analysis of Machine Learning

**DOI:** 10.3390/s21248192

**Published:** 2021-12-08

**Authors:** Taeyoon Kim, Soonchul Kwon, Yongju Kwon

**Affiliations:** Department of Civil and Environmental Engineering, Pusan National University, 2, Busandaehak-ro 63beon-gil, Geumjeong-gu, Busan 46241, Korea; rlaxodbs3@pusan.ac.kr (T.K.); zenga11@pusan.ac.kr (Y.K.)

**Keywords:** low-crested structure, machine learning, gradient boosting, wave attenuation, explainable artificial intelligence

## Abstract

The adoption of low-crested and submerged structures (LCS) reduces the wave behind a structure, depending on the changes in the freeboard, and induces stable waves in the offshore. We aimed to estimate the wave transmission coefficient behind LCS structures to determine the feasible characteristics of wave mitigation. In addition, various empirical formulas based on regression analysis were proposed to quantitatively predict wave attenuation characteristics for field applications. However, inherent variability of wave attenuation causes the limitation of linear statistical approaches, such as linear regression analysis. Herein, to develop an optimization model for the hydrodynamic behavior of the LCS, we performed a comprehensive analysis of 10 types of machine learning models, which were compared and reviewed on the prediction accuracy with existing empirical formulas. We found that, among the 10 models, the gradient boosting model showed the highest prediction accuracy with MSE of 1.0 × 10^−3^, an index of agreement of 0.996, a scatter index of 0.065, and a correlation coefficient of 0.983, which indicates a performance improvement over the existing empirical formulas. In addition, based on a variable importance analysis using explainable artificial intelligence, we determined the significant importance of the input variable for the relative freeboard (R_C_/H_0_) and the relative freeboard to water depth ratio (R_C_/h), which confirms that the relative freeboard was the most dominant factor for influencing wave attenuation in the hydraulic behavior around the LCS. Thus, we concluded that the performance prediction method using a machine learning model can be applied to various predictive studies in the field of coastal engineering, deviating from existing empirical-based research.

## 1. Introduction

Artificial structures for wave mitigation, such as breakwaters, headlands, detached breakwaters, and submerged breakwaters, are utilized to control coastal erosion problems by reducing incident wave energy and reducing sediment transports. Recently, shoreline deformation from beach erosion and scouring by coastal development has been rapidly increasing, along with sea level rise and external force of storm wave increases due to climate change [1]. Among these, coastal erosion and sedimentation caused by morphological change can lead to changes in the natural environment and ecosystem of coastal areas [2,3]. These problems directly/indirectly affect various factors involved in local economic activities in related field such as fishery and tourisms.

Low-crested submerged structures (LCS), such as detached breakwaters and artificial reefs, reduce the wave behind a structure according to the change in the freeboard at the still water level, thereby protecting onshore environment by inducing the wave [4]. Since the geometrical specifications of such LCS should be set under conditions to obtain the target wave transmission coefficient, the calculation or prediction of the transmission coefficient of a structure is an important factor in designing the structure. Various studies have been conducted to estimate the wave transmission coefficient of low-crested structures, and have proposed formulas for calculating the wave transmission coefficient [5,6,7]. However, most of the previously proposed formulas of wave transmission coefficients are estimated with assumptions and a regression analysis of mathematical experimental data, which inhibits the clear explanation of the natural phenomena. Existing models are limited to calculating wave transmission coefficients from a limited range of input parameters, resulting in less usefulness and applicability with low accuracy and reliability [8].

To cope with the limits, researchers have been conducting various studies to understand wave motion and wave characteristics around structures by applying numerical modeling [9,10] that are often used to model physical phenomena when analyzing hydrodynamic processes according to structural applications.

In order to simulate wave motion effectively and accurately, the non-hydrostatic effect must be considered. Ai et al. [11] simulated 3D free surface flow to suggest a new fully non-hydrostatic model. They verified the capabilities and numerical stability of the model through various test cases of surface wave motions. The proposed model presented the results of accurately and effectively resolving the motion of shortwaves with shoaling nonlinearity, dispersion, refraction, and diffraction. Ning et al. [12] used a fully nonlinear Boussinesq wave model to analyze runup characteristics according to solitary wave propagation conditions of fringing reefs. Furthermore, they presented results that reasonably reproduced the runup/rundown process for both non-breaking and breaking solitary waves through verification with laboratory experiment results. Hur et al. [13] analyzed hydrodynamic characteristics around permeable submerged breakwater using a 3D numerical scheme. They presented the detailed analysis results for distribution of wave height and wave breaking and average flow around the structure, considering the wave-structure-sandy seabed interactions in the 3D wave field. Recently, OlaFlow, an open source licensed open foam-based toolbox, has been developed. OlaFlow is composed of the Reynolds Average Navier Stokes equation and the conservation of mass equation, and the free water surface is tracked using Volume Of Fraction (VOF). Olaflow is widely used to analyze the hydrodynamic characteristics around structures in the field of coastal engineering [14].

Recently, a machine learning model has drawn attention to determine and to predict statistical structures from input/output data using a computer model. Machine learning is an inductive method as a field of artificial intelligence, which finds rules through learning using data and results, rather than a traditional program method that derives results through rules and data. It can easily solve complex engineering problems, and provide regression analysis of nonlinear relationships [15]. Compared to other traditional regression methods, it employs a specific algorithm that can learn from the input data itself and provides very accurate results on the output [16]. In machine-learning-based prediction models, various deep learning algorithms are employed, such as neural networks, decision trees, support vector machines (SVMs), and gradient boosting. In recent years, research using machine learning algorithms have been continuously increasing in the field of coastal engineering, in particular, for problems related to modeling behavior around coastal structures [17,18]. Kim et al. [19] used a neural network to estimate the stability of a breakwater, and presented the improved structure stability compared with the existing empirical formula. Based on the experimental data of Van Der Meer et al. [20], Koc et al. [21] proposed a model to predict the stability of a breakwater using a genetic algorithm.

Herein, to improve the feasible application of machine learning models in the field of coastal conservation engineering, we investigated the application of various machine learning models to predict the wave transmission coefficient of LCS. In addition, we proposed a machine learning pipeline model that selects a machine learning model suitable for data characteristics and performs an overall analysis of the model. Finally, we evaluated the applicability of the machine learning model by analyzing the accuracy and errors associated with the formulas for calculating the wave transmission coefficient of the existing LCS.

## 2. Methodology

### 2.1. Machine Learning Model

#### 2.1.1. Linear Regression Model

The linear regression model has the advantage that the parameters are linear and can be easily interpreted and analyzed quickly. Linear regression models were developed over 100 years ago and have been widely used over the past decades. However, a very restrictive shape results in low accuracy for data with nonlinear relationships. Linear regression creates a regression model using one or more characteristics and finds parameters w and b that minimize the mean squared error (MSE) between the experimental value (y) and predicted value ($\hat{y}$) (Equations (1) and (2)).

(1) $$\hat{y}=w\left[0\right]+x\left[0\right]\times x\left[1\right]+\cdots +w\left[p\right]\times x\left[p\right]+b$$

(2) $$\mathrm{MSE}=\frac{1}{n}\overset{n}{\underset{i=1}{\sum }}{\left(y_{i}-{\hat{y}}_{i}\right)}^{2}$$

#### 2.1.2. Lasso Regression

In the existing linear regression method, overfitting with poor predictive performance may occur when new data are provided. To solve this problem, a lasso regression was developed using L1 regulation to forcibly constrain the model (Equation (3)).

(3) $$E=\mathrm{MSE}+\mathrm{penalty}=\frac{1}{n}\overset{n}{\underset{i=1}{\sum }}{\left(y_{i}-{\hat{y}}_{i}\right)}^{2}+\alpha \overset{m}{\underset{j=1}{\sum }}\left|w_{j}\right|$$

Here, m is the number of weights, and α is a penalty parameter that determines w and b that minimize the sum of the MSE and penalty terms.

#### 2.1.3. Ridge Regression

Ridge regression is a model with an added L2 constraint to solve the overfitting problem of the linear regression model. The model not only fits the data of the learning algorithm, but also keeps the weights of the model as small as possible (Equation (4)).

(4) $$E=\mathrm{MSE}\;+\;\mathrm{penalty}=\frac{1}{n}\overset{n}{\underset{i=1}{\sum }}{\left(y_{i}-{\hat{y}}_{i}\right)}^{2}+\alpha \overset{m}{\underset{j=1}{\sum }}{\left|w_{j}\right|}^{2}$$

The weights become zero in lasso regression, whereas in ridge regression, the weights become close to zero but not zero. The difference is that if some of the input variables are important, lasso regression will have a higher accuracy, and if the importance of the input variables is similar overall, the ridge model will have a higher accuracy.

#### 2.1.4. SVM

The SVM was introduced by Boser et al. [22], inspired by the concept of statistical learning theory. The SVM is a method of finding a hyperplane composed of support vectors that can classify vectors of linearly different classes with the maximum margin for the distance between them [23]. The machine learning algorithm reflects data that cannot be classified linearly in a low-dimensional space in a high-dimensional space using a kernel function, and classifies it using a hyperplane. Representative types of kernel functions include polynomial, sigmoid, and radial basis function (RBF). In this study, the Gaussian RBF kernel was used and applied to the model [24].

#### 2.1.5. Gaussian Process Regression (GPR)

The GPR model is a probabilistic model based on nonparametric kernels. The Gaussian regression analysis model can be performed when the wave attenuation coefficient, which is the dependent variable, has a Gaussian shape [25]. Specifically, if a specific wave attenuation coefficient (K_t_^*^) is assumed as a random variable that includes an error, the expected wave attenuation coefficient with the error removed can be expressed as a covariance function between the mean and the error (K_t_^*^= K_t_ + ε). Assuming that this error covariance can be interpreted as a kernel function, Bayesian analysis model can predict wave attenuation characteristics [26].

#### 2.1.6. Ensemble Method

The ensemble method was created to improve the performance of the classification and regression tree (CART). The method creates a more accurate prediction model by creating several classifiers and combining their predictions. In other words, the method derives a highly accurate prediction model by combining several weak classifier models, and not using a single strong model. Ensemble models can be broadly divided into bagging and boosting models. Bagging method reduces variance by using average or voting methods for the results predicted by various models, and boosting method synthesizes weak classifiers into strong classifiers. In this study, we performed a predictive study using boosting and random forest (RF) ensemble methods.

(1) Random Forest (RF)

RF is a method that is employed to improve defects such as the variance and the performance fluctuation range of the decision tree being large. RF combines the concept and properties of bagging with randomized node optimization to overcome the shortcomings of existing decision trees and improve the generalization performance. In line with bagging method, the process of extracting bootstrap samples and creating a decision tree for each bootstrap is similar, but instead of selecting the optimal partition within all predictors for each node, the RF randomly extracts predictors and creates optimal partitions within the extracted variables [27]. In other words, RF creates several learners of low importance since it determines slightly different training data through bootstrap to give maximum randomness, and simultaneously combines the randomization of predictors. Important hyperparameters of RF include max_features, whether to use bootstrap, and n_estimator. The max_features parameter represents the maximum number of features to be used in each node, bootstrap is the option to allow duplication in data sampling conditions for each classification model, and n_estimator means the number of trees to be created in the model [28].

(2) Boosting method

Boosting is a method that is used to create strong classifiers from a few weak classifiers, and is a model created by further boosting the weights on the data at the boundary. AdaBoost is the most common and widely used ensemble learning algorithm, and is specifically one of the boosting families of ensemble learning. The main feature of AdaBoost is that after generating a weak classifier using initial training data, the distribution of the training data is adjusted according to the prediction performance dependents on the weak classifier training. The weight of the training sample with low prediction accuracy was increased by using the information received from the classifier in the previous stage. In other words, the method improves learning accuracy by adaptively changing the weights of samples with a low prediction accuracy in the previous classifier. The method combines these weak classifiers with low prediction performance to create a strong classifier with slightly better performance. Gradient boosting method, which is applied in this study, also sequentially adds multiple models in the same way as the Adaboost model [29]. The biggest difference between the two algorithms is the recognition of weak classifiers. While AdaBoost recognizes values that are more difficult to classify by weighting them, Gradient Boost uses a loss function to classify errors. In other words, the loss function is an indicator that can evaluate the performance of the model in learning specific data, and the model result can be interpreted differently depending on which loss function is used.

### 2.2. Analysis of Machine Learning Model

#### 2.2.1. Performance Measurement

The correlation coefficient, which indicates the correlation between the predicted output value and the measured value of the model, is an important factor for evaluating the predictive performance of a machine learning model. To analyze the predictive performance of the model, in this study, we measured the performance of the model using the mean square error (MSE), index of agreement (I), scatter index (SI), and R^2^, which represent the correlation coefficient. For each dataset, the correlation coefficient between the experimental and predicted values is as shown in Equations (5)–(8).

(5) $$\mathrm{MSE}=\frac{\sum_{i=1}^{n}{\left(x_{i}-{\;y}_{i}\right)}^{2}}{n}$$

(6) $$I=1-\frac{\sum_{i=1}^{n}{\left(y_{i}-{\;x}_{i}\right)}^{2}}{\sum_{i=1}^{n}{\left(\left|y_{i}-\bar{x}\right|+\left|x_{i}-\bar{x}\right|\right)}^{2}}$$

(7) $$\mathrm{SI}=\frac{\sqrt{\frac{1}{n}\sum_{i=1}^{n}{\left(x_{i}-{\;y}_{i}\right)}^{2}}}{\bar{x}}$$

(8) $$R^{2}=1-\frac{\sum_{i=1}^{n}{\left(x_{i}-{\;y}_{i}\right)}^{2}}{\sum_{i=1}^{n}{\left(x_{i}-\bar{y}\right)}^{2}}$$

Here, $x_{i}$ and $y_{i}$ are the experimental and predicted values, respectively, $\bar{x}$ and $\bar{y}$ are the mean values of the experimental and predicted values, respectively, and n is the sample number. Statistically, the closer R^2^ and I are to 1 and the smaller the MSE and SI, the higher is the reliability.

#### 2.2.2. Analysis Method of Feature Importance

(1) eXplainable Artificial Intelligence (XAI)

XAI was developed to help users understand the overall characteristics of how an AI system works and correctly interprets the final result. XAI is a surrogate model that makes possible to explain the process of calculating results for the correlation between input variables and dependent variables by determining the major factors that affect the prediction of a machine learning model. The interpretation of such a machine learning model is an important analysis method for deriving a suitable learning model according to various conditions or to increase the prediction stability of the model through quantitative analysis of predicted values through input variables.

The analysis method of the artificial intelligence system analyzes the characteristics of input variables to interpret the model learning and prediction process, and is divided into global and local interpretations. Global interpretation is a method of interpreting the overall analysis process and results of a model, and local interpretation is a method of interpreting model predictions for a single observation or part of a data set, and interpreting the results derived from the model for one specific input data.

(2) Shapley Additive exPlanations (SHAP)

The Shapley value is the mean value of the marginal contribution for all possible sets to understand the importance of one characteristic based on game theory (theorizing about what decisions or actions each other takes in situations where multiple themes influence each other). Lundberg et al. [30] developed the SHAP machine analysis model, which achieves the highest accuracy, with a solid theoretical background among the machine learning analysis models that have been released to date, and the Shapely value is given in Equation (9).

(9) $$\emptyset_{i}=\underset{S\in N/\left(i\right)}{\sum }\frac{\left|S\right|!\left(n\;-\left|S\right|-1\right)!}{n!}(f\left(S\cup^{\;}\left\{i\right\}-\;f\left(S\right)\right)$$

Here, S is a subset of the features used in the model, {i} is the vector of feature values of observations for explanation, n is the number of characteristics. f(S) is the value obtained by subtracting the predicted value from one observation from the average predicted value obtained from the data for a combination of feature values.

SHAP values are obtained by using the conditional expected value function of the machine learning model for Shapley values. The Shapley values for all input features are obtained, and the SHAP values can be interpreted locally and globally with the SHAP mean for each feature for each observation. The input feature importance can be expressed by visualizing the input feature based on the dataset through the average or sum of the absolute values of the SHAP values. In the case of the partial dependence plot provided by SHAP, the value of the input characteristic of each instance and the corresponding SHAP values are expressed as dots for all instances, and the average of the predicted values is calculated by changing the specific characteristic value of each instance. In this study, we analyzed the characteristics of a machine learning model built using the SHAP model.

### 2.3. Empirical Formula of Wave Transmission Coefficient

**Figure 1:**
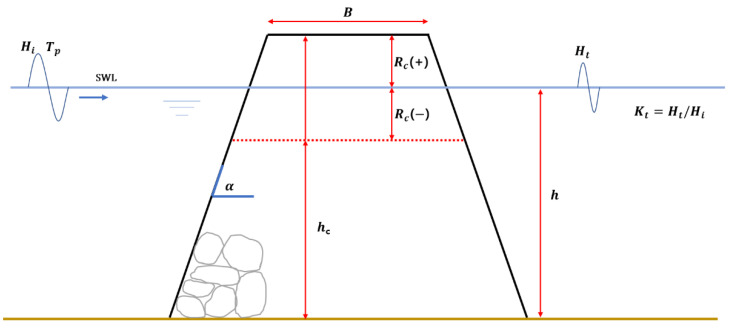
Example of a cross-section of a low-crested and submerged breakwater and the governing parameters.

The wave transmission coefficient represents the ratio of the incident wave height before passing through the LCS and the average wave height after passing through the LCS (Figure 1).

Existing theoretical and empirical equations for the LCS are based on the experimental results of the hydraulic model, and many researchers have proposed empirical equations to predict the wave transmission coefficient using experimental data [31,32,33].

The suggested equation for the wave transmission coefficient by D’Angremond et al. [32] is as follows (Equations (10) and (11)):

(10) $$K_{t}=-0.4\frac{R_{c}}{H_{i}}+0.64{\left(\frac{B}{H_{i}}\right)}^{-0.31}\left(1-{\;e}^{-0.5\xi }\right),\;\frac{B}{H_{i}}<8$$

(11) $$K_{t}=-0.35\frac{R_{c}}{H_{i}}+0.51{\left(\frac{B}{H_{i}}\right)}^{-0.65}\left(1-{\;e}^{-0.41\xi }\right),\;\frac{B}{H_{i}}>12$$

Here, $R_{c}$ is the crest freeboard, $H_{i}$ is the incident wave height, B is the crown width ξ is the surf similarity coefficient for breakwater (ξ=tanα/HiL0). However, in the aforementioned equation, the effective range of the wave transmission coefficient is limited to 0.075–0.8. Van der Meer [31] suggested a wave transmission coefficient equation based on the breakwater coefficient to improve the accuracy of the wave transmission coefficient (Equations (12) and (13)).

(12) $$K_{t}=-0.3\frac{R_{c}}{H_{i}}+0.75\left(1-{\;e}^{-0.5\xi }\right),\;\;\xi <3$$

(13) $$K_{t}=-0.3\frac{R_{c}}{H_{i}}+0.75{\left(\frac{B}{H_{i}}\right)}^{-0.31}\left(1-{\;e}^{-0.5\xi }\right),\;\;\xi >3$$

Bleck and Oumeraci [33] proposed the exponential decay equation of the wave transmission coefficient of the LCS, according to the relative freeboard (Equation (14)).

(14) $$K_{t}=1-0.83e^{0.72\frac{R_{c}}{H_{i}}}$$

In previous studies, factors such as relative freeboard $\left(\frac{R_{c}}{H_{i}}\right)$, relative crest width ($\frac{B}{H_{i}}$), front slope of structure (tanα), and wave steepness (HiL0), are classified as the factors related to wave decay around the LCS, and the empirical formula for this is presented. Figure 1 shows cross-section of LCS structure. Herein, the crest freeboard ($R_{C}=h_{c}-\;h$) indicates the differences between structure depth and water depth, which has a positive value in emerged state and negative value in submergence state in still-water level.

In this study, we compared and reviewed the results of calculating the wave transmission coefficient using the existing empirical formula (Equations (10)–(14)), and the prediction results using a machine learning model.

### 2.4. Model Design Condition and Method

#### 2.4.1. Machine Learning Automatic Pipeline Model

In this study, we applied 10 machine learning models, namely linear regression, kernel ridge (KR), ridge, lasso, GPR, SVM, RF, artificial neural network (ANN), gradient boosting regressor (GBR), and AdaBoost, to compare and review the performance dependencies on the characteristics of each model. To determine the optimal conditions of the automatic pipeline model dependent on input data characteristics, we adjusted hyperparameters using Grid-searchCV, and constructed automatic models for 10 machine learning models using the scikit-learn pipeline. The optimal machine learning model, selected through the automatic model, was analyzed to determine the importance of variables affecting the wave control of LCS using the machine learning analysis package SHAP.

#### 2.4.2. Machine Learning Model Configuration and Input Conditions

The 260 items of input data were obtained and applied in this study, with reference to the results of hydraulic model experiments with existing LCS by Seelig [34], Daemrich and Kahle [35], van der Meer [20], and Daemen [36]. Data on the wave transmission coefficient were obtained from DELOS database for permeable structures. The 260 data consist of:

- 81 data on rubble mound emerged/submerged breakwater [Seelig];
- 95 data on tetrapod submerged breakwater [Daemrich and Kahle];
- 31 data on rubble mound emerged/submerged breakwater [Van der Meer];
- 53 data on rubble mound emerged/submerged breakwater [Daemen].

In the data applied to the model, wave attenuation characteristics behind the structure were analyzed using random wave, and the data in range of 0.021 to 0.231 m were applied to wave height, and 0.91–3.66 s to wave period (Table A1). Through various studies, research results on the wave attenuation mechanisms of LCS and various factors that have a dominant influence on wave attenuation are presented. Van der Meer [31] proposed a wave transmission coefficient equation using R_c_/H_0_, B/H_0_ and ξ. Shin et al. [37] suggested the wave transmission coefficient equation using B/L_0_, R_c_/h, and Gadomi et al. [38] performed research on porosity and h_c_/h. Therefore, in this study, we used seven dimensionless numbers (X = {X_1_, X_2_,...., X_7_}) as input variables (Table 1) based on previous studies. Here, R_c_/H_0_ is the relative freeboard, B/H_0_ is the relative crest width, ξ is the surf similarity parameter, B/L_0_ is the ratio of the crest width to the wavelength, R_c_/h is the relative freeboard to water depth ratio, Dn_50_/h_c_ is the ratio of the nominal diameter to the crest height, and h_c_/h is the relative structure height. Herein, D_n50_ means nominal diameter, which is the ratio of median mass of unit (M_50_) and $\mathrm{mass}\;\mathrm{density}\;\mathrm{of}\;\mathrm{the}\;\mathrm{rock}\;\left(\rho_{r}\right)$ (Dn50=(M50ρr). Therefore, Dn_50_/h_c_ parameter is a factor related to the effect of voids as the ratio of the structure height to the nominal diameter. Surf similarity parameter (ξ=tanα/HiL0) represents the ratio of the front slope (tanα) and wave slope (HiL0), which is an important parameter in relation to wave breaking. The front slope of the structure applied in this study was in the range of 1:1.38–1:4, and various slope conditions were considered.

**Figure 2:**
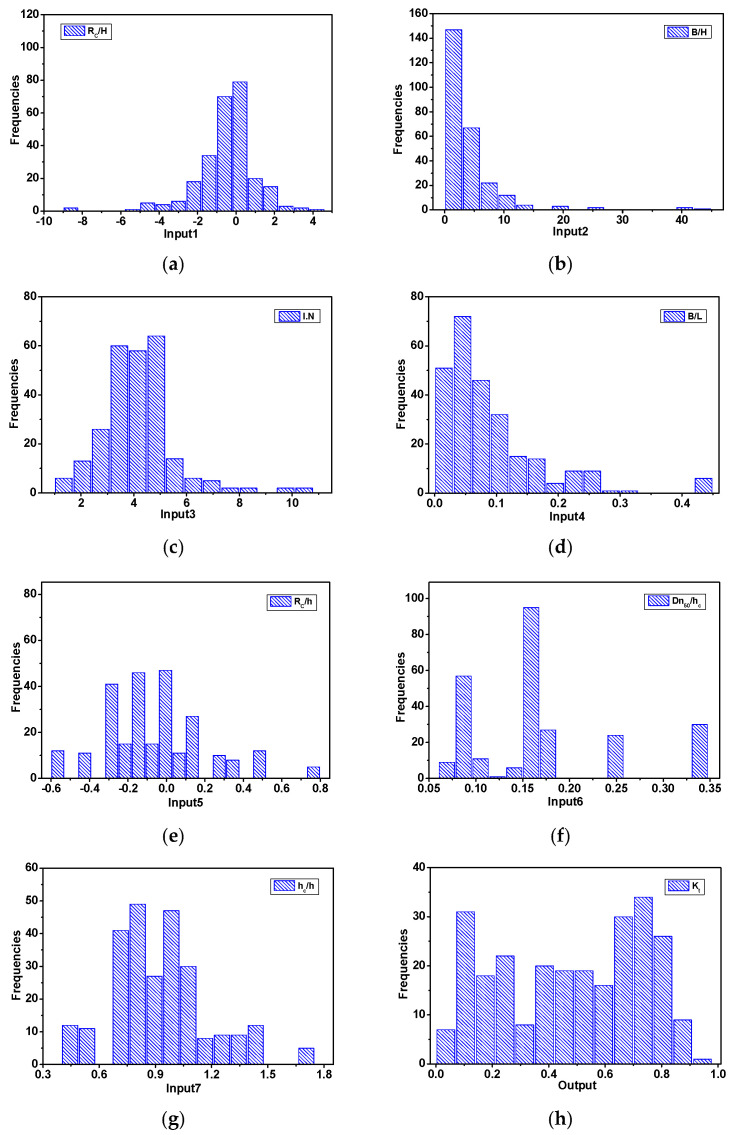
Results and discussion. (**a**): R_C_/H_0;_ (**b**): B/H_0;_ (**c**): ξ; (**d**): B/L_0;_ (**e**): R_C_/h; (**f**): Dn_50_/h_c_; (**g**): h_c_/h; (**h**): K_t_.

Figure 2 depicts the statistical distributions of the input and output variables. To reflect the same feature scale, the input variable was converted to a range of 0 to 1 using max-min normalization.

## 3. Results and Discussion

### 3.1. Comparison of Machine Learning Model and Model Selection

**Figure 3:**
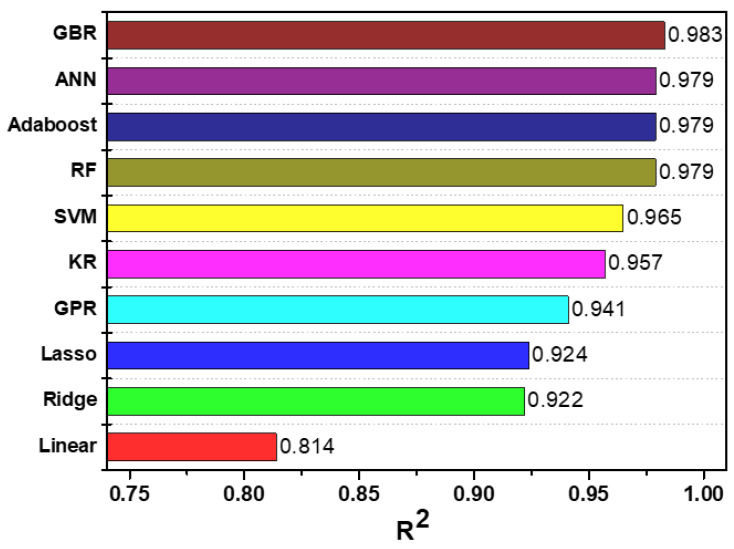
Performance analysis of machine learning models.

**Figure A1:**
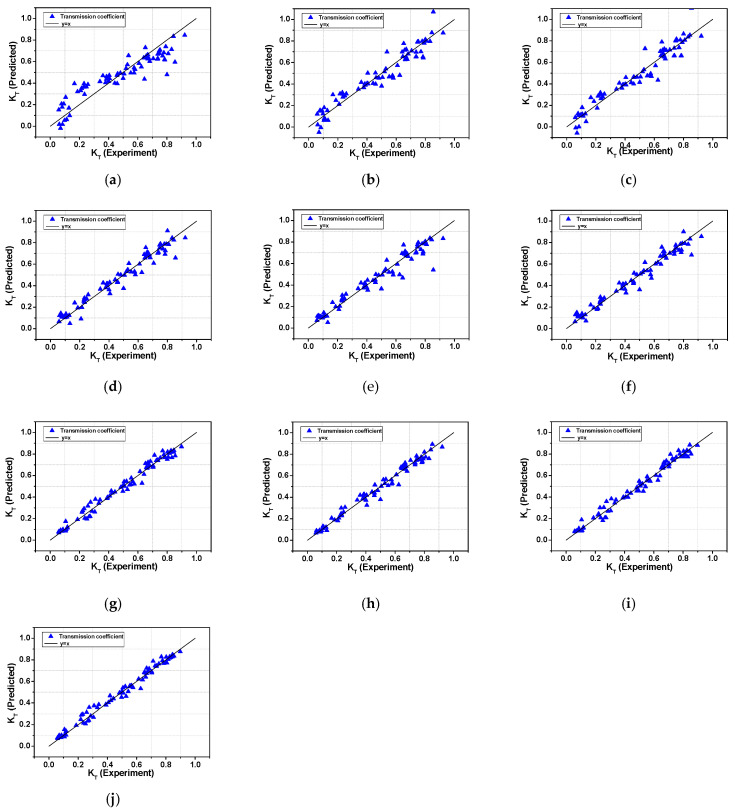
Measured versus predicted values for the various models. (**a**) Linear; (**b**) Ridge; (**c**) Lasso; (**d**) KR; (**e**) GPR; (**f**): SVM; (**g**) RF; (**h**) ANN; (**i**) AdaBoost; (**j**) Gradient boost.

Recently, research on the development and application of various machine learning techniques has been performed in the field of computer science. The performance of these machine learning models differs according to the characteristics of the input variables. Therefore, to model the fluid mechanical behaviors of the LCS, we analyzed the performance of the model by using 10 linear and nonlinear regression models. Figure 3 presents the performance results of the machine learning model of the artificial coral reef data derived from the machine learning pipeline model. Among the 10 machine learning models, GBR showed the highest model performance with an R^2^ = 0.983, and the linear regression method showed the lowest performance with an R^2^ = 0.814. Table 2 shows the model performance results based on the application of the 10 machine learning methods. The ensemble method (Adaboost, GBR, and RF), including the ANN method, showed the highest model accuracy (under 1.3 × 10^−3^ MSE) and model performance (>0.979 of R^2^). Among them, GBR yielded the highest model prediction accuracy. This indicates that the boosting method reinforces the weak classifier. In addition, the linear regression models (linear, ridge, and lasso) showed low accuracy, indicating that the application of the linear model cannot reflect the nonlinear characteristics of the data. Furthermore, it is believed that the linear model shows low model prediction performance when the model has nonlinearity between the input variable and the dependent variable. However, the performance of the model could be increased by regulating the L_1_ and L_2_ weights. We presented the wave transmission prediction results for the LCS using a machine learning model, as shown in Figure A1, which shows the distribution of the experimental values and prediction values of the test set. In terms of designing coastal structure, the estimation of the wave transmission coefficient with high accuracy is most important. As a result of comparing machine learning models, the GBR model shows the highest accuracy in terms of predicting the wave transmission coefficient for LCS structure. Therefore, we performed an analysis by applying the GBR model, which showed the highest accuracy in predicting the hydraulic characteristics around the LCS.

### 3.2. Model Performance Analysis

#### 3.2.1. Results of Splitting a Dataset

To determine the most accurate parameter of the GBR model, we divided the collected data into training data and test data. Traditionally in machine learning, when the number of data is small, the training data and test data are divided by 7:3; however, recently, when the number of data is large, the dataset can be divided by 9:1. We divided the data set into 7:3, 8:2, and 9:1 conditions to perform sensitivity analysis on model accuracy. Table 3 shows the model performance results according to data splitting condition, and the highest R^2^ can be obtained under the conditions of 9:1 and 8:2. As the training set ratio increases, the number of training data increases, which allows the GBR model to produce a strong learner. However, since overfitting of the model and generalization of the model may be difficult due to insufficient data in the test set under 9:1 condition, we built the model by applying the 8:2 condition.

**Figure 4:**
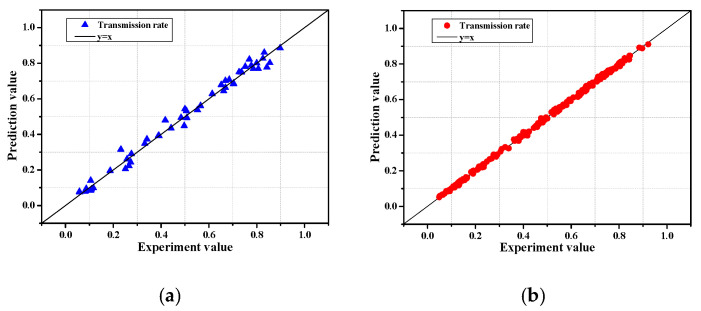
Distribution of experimental and predicted values according to the GBR model application. (**a**): Test set. (**b**): Training set.

Figure 4 shows the prediction results of the training data and test data considering seven input variables using gradient boosting; the horizontal axis represents the experimental values, and the vertical axis represents the distribution of predicted values. As for the results, I was 0.999, SI was 0.032, and R^2^ was 0.999 for the training data set, and the MSE was 0.8 × 10^−3^, I was 0.997, SI was 0.058, and R^2^ was 0.988 for the test data set, indicating the excellent prediction performance for the wave transmission coefficient. As a result, it is deemed that the performance prediction method using such a machine learning model can be applied to various predictive studies in the field of coastal engineering, deviating from existing empirical-based research.

#### 3.2.2. 10-Fold Validation Analysis

**Figure 5:**
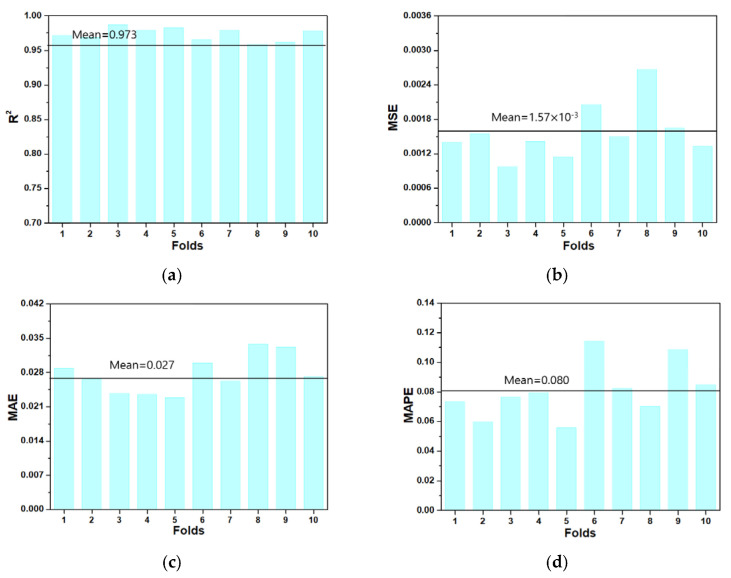
Results for 10-fold cross validation. (**a**) R^2^; (**b**) MSE; (**c**) MAE; (**d**) MAPE.

To verify the model performance of the GBR model, we utilized 10-fold cross-validation. This method was developed to minimize the bias associated with random sampling of the training set. The entire data sample was divided into 10 parts: nine were used for training, and one was used for model validation. The process of cross-validation was performed ten consecutively. The 10-fold cross-validation method ensured the generalization and reliability of the model performance. Figure 5 shows the model performance results obtained using the 10-fold cross-validation method. Figure 5a shows the results of R^2^ according to each fold and shows slight fluctuations; however, the minimum and maximum values were 0.958 and 0.987, respectively. Figure 5b shows the minimum value of 0.97 × 10^−3^, and the maximum value of 2.70 × 10^−3^ for MSE, showing that all errors are minimal, and a high level of accuracy is maintained.

Table 4 shows the model performance and statistical information using the 10-fold cross-validation method. The mean R^2^ is 0.973, and the std is 0.009, demonstrating that the results have a small deviation. In addition, the MAE and MAPE are 0.027 and 0.080, respectively, indicating small prediction errors.

#### 3.2.3. Evaluation of GBR Model Using Another Data Set

To verify the generalization of developed model additionally, we applied new data set to the model. For the verification for the GBR model, we used 41 data sets from Delft Hydraulics [39] and Allsop [40] as data.

- 20 data on rubble mound LCS (submerged) [Delft Hydraulics].
- 21 data on rubble mound LCS (emerged) [Allsop].

**Figure 6:**
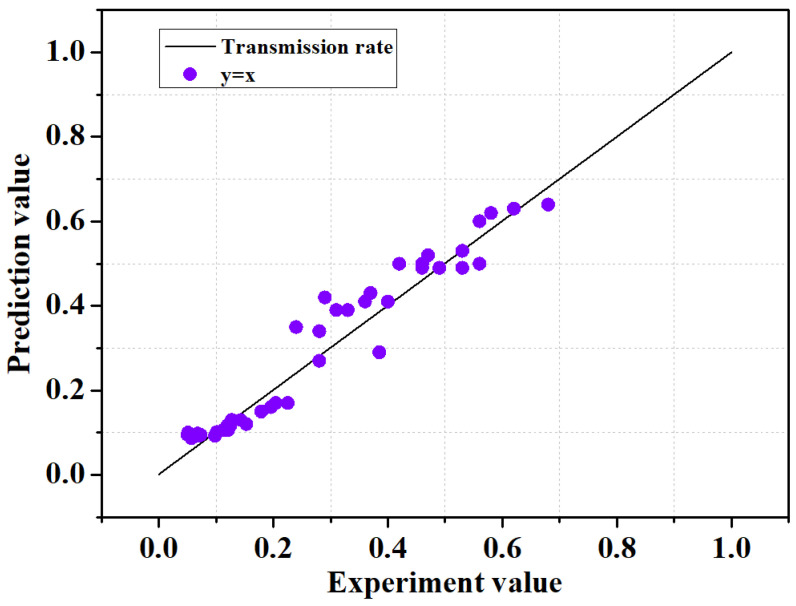
Results for the new data set.

Figure 6 Shows the prediction results for new data set using GBR model. For the new data set, the relationship between the predicted and tested values converges closely to the ideal y = x line, which shows the good prediction results of the proposed model. Quantitative performance measurement showed high accuracy with R^2^ = 0.93, MSE = 2.3 × 10^−3^, I = 0.98, SI = 5.2 × 10^−3^, which suggests that the GBR model actually has high accuracy in predicting the wave transmission coefficient of LCS even for completely new data sets.

#### 3.2.4. Feature Importance Analysis

**Figure 7:**
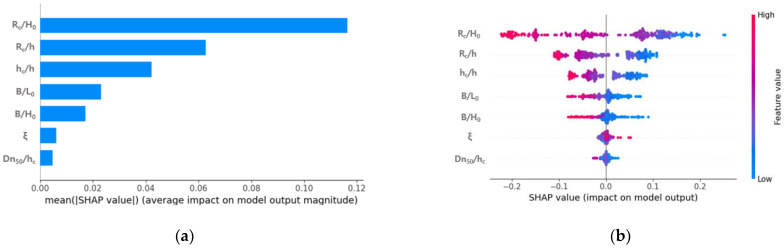
Analysis of feature importance. (**a**): SHAP feature importance. (**b**) Summary plot (feature effects).

Around the coastal structures, the wave attenuation effect is not an action that is independent of the input variables, but rather a complex interaction dependent on the variables. Thus, the relative importance of each variable in the model unit to the total observations should also be analyzed. Since the importance of the input variable is a measure of how much the variable affects the dependent variable, analysis for the correlation between the input and dependent variables is important. Therefore, we analyzed the importance of variables that affect the wave attenuation of the LCS. Figure 7 shows the importance of input variables that affect the dependent variable (wave transmission coefficient) when applying the 260 hydraulic model experiment results to the GBR model. Figure 7a shows the variable importance, and the x-axis represents the average of the absolute values of the Shapley values of the input variables throughout the data. In short, this means that the average influence of the input variable on the dependent variable, and the larger the x-axis value, the greater the influence on wave attenuation.

As a result of the variable importance analysis, the SHAP value of relative freeboard (R_c_/H_0_) was 0.116, which verifies that R_c_/H_0_ was the most dominant parameter for wave control and wave energy reduction in hydrodynamics behavior around the LCS. Next, the relative freeboard to water depth ratio (R_c_/h) and relative structure height (h_c_/h) were 0.062, 0.042, respectively, and the results show that the input variable related to the freeboard has a dominant influence over 80% of the total wave height attenuation behind the structure. The freeboard should be prioritized for wave control in the design of the structure, as the attenuation effect of wave energy passing over the LCS along with wave breaking increases as the freeboard increases. The SHAP values of the ratio of the crest width to wavelength (B/L_0_) and relative crest width (B/H_0_) were 0.023 and 0.017, respectively, indicating that the input variable related to the crest width has an effect of more than 8.6% of the total on the wave attenuation. Figure 7b shows a summary plot combining the feature importance and feature effects of the input variables. Here, they are arranged in order of importance, so that the one with the highest feature importance is placed at the top. The stronger the red shading of the corresponding feature value, the more positive is the influence on the wave transmission coefficient (K_t_), and the stronger the blue shading, the more negative is the influence. As a result, as R_c_/H_0_, R_c_/h, h_c_/h, B/L_0_, and B/H_0_ increased, the wave transmission coefficient decreased, and as the surf similarity coefficient (ξ) increased, the wave transmission coefficient tended to increase. The results showed that this sensitivity trend was in line with engineering practice and physical background.

#### 3.2.5. Influence of Input Variable Number

**Figure 8:**
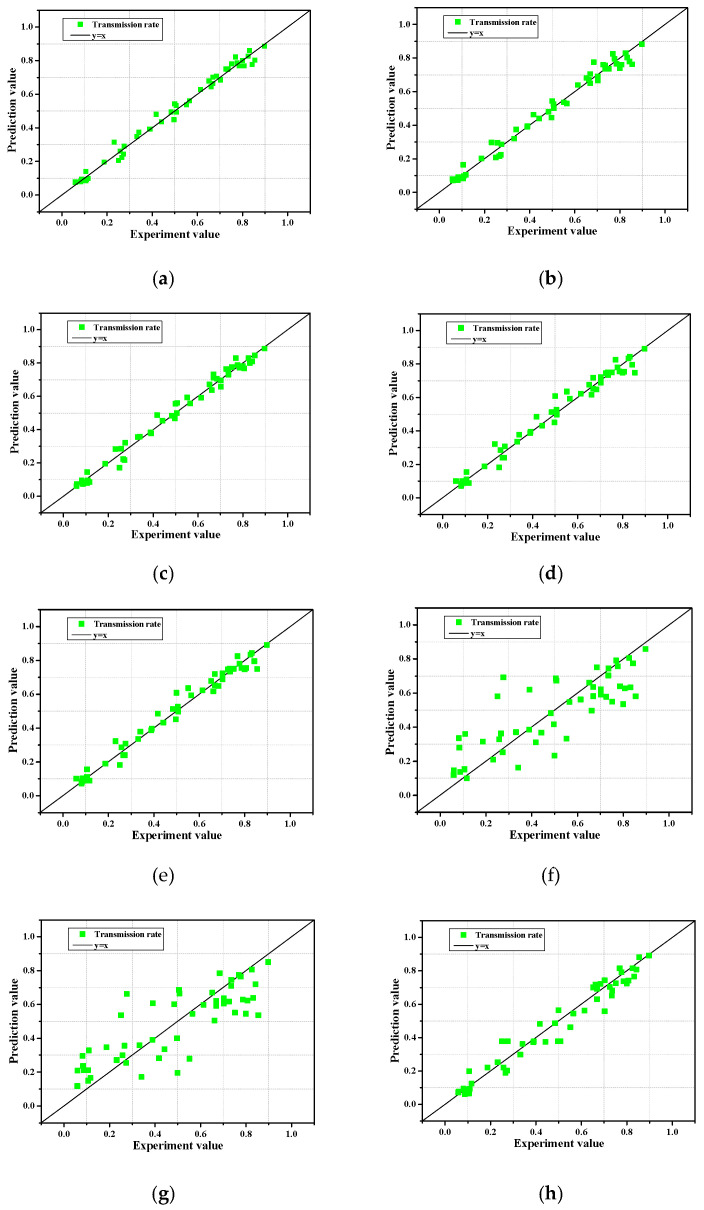
Predicted and experimental values for the eight combinations. (**a**): Combination 1; (**b**): Combination 2; (**c**): Combination 3; (**d**): Combination 4; (**e**): Combination 5; (**f**): Combination 6; (**g**): Combination 7; (**h**): Combination 8.

In this study, we analyzed the model accuracy by applying an input variable consisting of seven dimensionless numbers (X = {X_1_:R_c_/H_0_, X_2_:B/H_0_, X_3_:ξ, X_4_:B/L_0_, X_5_:R_c_/h, X_6_:Dn_50_/h_c_, and X_7_:h_c_/h}). If it is possible to build a model with high accuracy by excluding insignificant input variables and constructing a model with only important input variables, it is also possible to reduce computational complexity and to derive good results in terms of time efficiency. Accordingly, we analyzed the effects on model performance when some input variables or data were not reflected through various combinations. Table 5 presents the model performance results based on the eight combinations of input variables, and Figure 8 presents the results of the predicted values and experimental values for the eight combinations.

Combination 1 showed the model performance results when applying pristine seven dimensionless input variables, indicating the highest accuracy with an MSE of 0.8 × 10^−3^, and R^2^ of 0.988. In contrast, combination 7, which applied four input variables (X_2_:B/H_0_, X_3_: ξ, X_6_:Dn_50_/h_c_) showed the lowest accuracy with an MSE of 22.9 × 10^−3^, R^2^ of 0.668. In addition, combination 8, which applied three input variables (X_1_:R_c_/H_0_, X_5_:R_c_/h, X_7_:h_c_/h), showed relatively high accuracy with an MSE of 3.7 × 10^−3^, R^2^ of 0.947, despite the application of a small number of input variables. It is worth noting that the accuracy of the model does not simply increase with an increase in the number of input variables, as can be seen from the combination 1–8 results. In addition, in combination 2, the relative freeboard (X_1_:R_c_/H_0_), which was classified as the most important factor in the sensitivity analysis, was not taken into account; however, combination 2 had a relatively high accuracy with an MSE of 1.3 × 10^−3^ and an R^2^ of 0.981. Even if the relative freeboard (X_1_:R_c_/H_0_) is ignored, it is judged that combination 2 showed high accuracy by considering the factors (X_5_:R_c_/h, X_7_:h_c_/h) related to the freeboard. However, combinations 6–7, which ignored the factors related to the crest height (X_1_:R_c_/H_0_, X_5_:R_c_/h, X_7_:h_c_/h), showed low accuracy. In summary, the factors related to the freeboard (X_1_:R_c_/H_0_, X_5_:R_c_/h, X_7_:h_c_/h) are the most important input variables to consider when obtaining predictions with high accuracy.

### 3.3. Comparison of Wave Transmission Coefficient Using Empirical Formulas and Machine Learning Models

**Figure 9:**
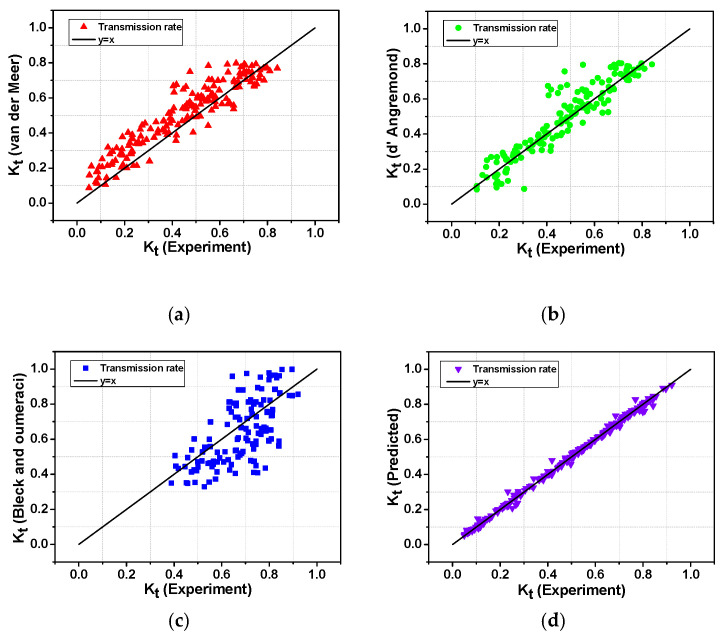
Comparison of predictive performance of empirical formulas and machine learning model. (**a**): Comparison of wave transmission formulas (Van der Meer) with experimental data. (**b**) Comparison of wave transmission formulas (D’Angremond) with experimental data. (**c**) Comparison of wave transmission formulas (Bleck and Oumeraci) with experimental data. (**d**) Comparison of GBR model with experimental data.

Figure 9a,b show the results of substituting all experimental data into the empirical formula for the wave transmission coefficient of LCS at the low-crest submerged breakwater suggested by Van der Meer [31] and D’Angremond [32]. However, the results out of the effective range (0.075 < K_t_ < 0.8) suggested by the empirical formula were excluded. As a result of the prediction of the wave transmission coefficient using the Van der Meer empirical equation, the MSE was 0.009, and the determination coefficient (R^2^) was 0.81, indicating that the overall result of the empirical formula was overestimated, with respect to the experimental value (Figure 9a). As a result of the prediction of the wave transmission coefficient using the D’Angremond empirical formula, the MSE was 0.006 and the correlation coefficient (R^2^) was 0.84, showing fewer errors than the Van der Meer empirical formula and high prediction accuracy (Figure 9b). However, in the case of empirical formulas proposed by Van der Meer [31] and D’Angremond [32], applicable formulas are classified according to the surf similarity parameter (ξ=tanα/HiL0) and the relative crest width (B/H_0_), and the effective range of the wave transmission coefficient is limited to 0.075 < K_t_ < 0.8, which has a disadvantage in that uncertainty increases for other ranges. Figure 9c shows the results of substituting all experimental data into the empirical formula for the wave transmission coefficient of a low-crest submerged breakwater proposed by Bleck and Oumeraci [33]. In the case of the prediction results for the wave transmission coefficient using the empirical formula, the MSE was 0.017, and the determination coefficient (R^2^) was 0.71, indicating that the result of the empirical formula showed a low similarity to the experimental value overall. In addition, for the experimental value under 0.32, the transmission coefficient was 0.170, with a low prediction accuracy. The wave transmission coefficient of the LCS should consider the influence of various factors, such as crown depth, crown width, and porosity; however, the experimental formula of Bleck and Oumeraci [33] only considered the relative freeboard (R_C_/H_0_), which led to a lower prediction accuracy than the other empirical formulas. Table 6 shows the results of comparing the statistical indicators of the existing empirical formula and the GBR model. Overall, all statistical indicators showed that the results of the boosting model showed higher prediction accuracy than that of the existing empirical formula. Furthermore, unlike the existing empirical formula, the boosting model does not need to set the effective range of the wave transmission coefficient, and does not require a separate formula dependent on the input variable (Figure 9d). In summary, a very accurate wave transmission coefficient can be predicted by inputting the seven input variables required by the machine learning model. This is because machine-learning models can interpret the non-linear relationships between independent and dependent variables. In the case of the empirical formula, analysis is possible only in the effective range of the wave transmission coefficient, whereas when the GBR model is applied, it shows good predictive performance in all ranges.

## 4. Conclusions

In this study, we investigated the hydrodynamic performance modeling of a low-crested structure using 10 machine learning models, including linear and non-linear models. To construct the model, we used 260 hydraulic model test data for training (80%) and prediction (20%). To predict the wave transmission coefficient behind the structure, we applied seven dimensionless parameters (R_C_/H_0_, B/H_0_, ξ, B/L_0_, R_C_/h, Dn_50_/h_c_, and h_c_/h). In addition, we evaluated the correlation between the input variable and dependent variable by analyzing the main factors that affect the prediction of machine learning models using XAI. The wave transmission coefficient for the linear model (M8, M9, and M10) among the machine learning models showed low prediction accuracy; however, the ensemble technique, the GBR model (M2) in particular, showed the highest accuracy to predict the wave transmission coefficient of a structure with a given input variable. To validate the machine learning models, we performed a 10-fold cross-validation, which indicates that the resulting R^2^ was 0.973, and the mean MAPE was 2.7%, confirming a significantly low prediction error. This small degree of error proves the generalization of the model reasonably. Based on the sensitivity analysis, we confirmed that the input variable for the relative freeboard (R_C_/H_0_), and the relative freeboard to water depth ratio (R_C_/h) show that the importance of independent variables is significant. As a result, freeboard was found to be the most dominant factor influencing wave attenuation in the hydraulic behaviors around the LCS. In addition, we comprehensively analyzed the results of the empirical formulas and machine learning models. In the wave transmission prediction of the trained gradient boosting model, the MSE was 0.8 × 10^−3^, I was 0.997, SI was 0.058, and R^2^ was 0.988, which indicates high prediction accuracy and improved wave transmission coeffcient prediction performance, compared with existing empirical results. Since the prediction using machine learning can perform analysis non-linearly, the wave transmission coefficient of a LCS can be predicted precisely and efficiently, in contrast to the regression method adopted by the exiting empirical formula. It is determined that the constructed machine learning automated pipeline model can be utilized for not only wave attenuation studies on LCS, but also various applications in coastal engineering.

## Figures and Tables

**Table 1:** Definitions and ranges of scaled model parameter.

Parameter		Definition	Average	Max	Min
X_1_	R_C_/H_0_	Relative freeboard	−0.494	4.0	−8.696
X_2_	B/H_0_	Relative crest width	4.525	43.478	0.889
X_3_	ξ	surf similarity parameter	4.145	10.541	1.181
X_4_	B/L_0_	Ratio of the crest width to wave length	0.09	0.424	0.012
X_5_	R_C_/h	the relative freeboard to water depth ratio	−0.065	0.734	−0.56
X_6_	Dn_50_/h_c_	Ratio of the nominal diameter to structure height	0.166	0.336	0.065
X_7_	h_c_/h	The relative structure height	0.935	1.734	0.44
Y	K_t_	Transmission coefficient	0.482	0.922	0.049

**Table 2:** Proposed machine learning regressors and the resulting attenuation coefficient.

	Performance Measures			
ML Regressor	MSE	I	SI	R^2^
M1. AdaBoost	0.0013	0.995	0.073	0.979
M2. Gradient boost	0.0010	0.996	0.065	0.983
M3. ANN	0.0013	0.995	0.078	0.979
M4. RF	0.0013	0.995	0.073	0.979
M5. SVM	0.0022	0.991	0.101	0.965
M6. GPR	0.0038	0.984	0.132	0.941
M7. Lasso	0.0049	0.981	0.150	0.924
M8. Ridge	0.0050	0.980	0.152	0.922
M9. KR	0.0028	0.989	0.113	0.957
M10. Linear	0.0121	0.944	0.235	0.814

**Table 3:** Performance of machine learning model according to splitting a dataset.

Splitting Ratio	MSE	I	SI	R^2^
7:3	1.0 × 10^−3^	0.996	0.065	0.983
8:2	0.8 × 10^−3^	0.997	0.058	0.988
9:1	0.8 × 10^−3^	0.997	0.051	0.988

**Table 4:** Performance of 10-fold cross validation.

Folds	Performance Measures			
	MSE	R^2^	MAPE	MAE
Fold 1	1.4 × 10^−3^	0.971	0.073	0.029
Fold 2	1.5 × 10^−3^	0.969	0.060	0.027
Fold 3	1.0 × 10^−3^	0.987	0.076	0.024
Fold 4	1.4 × 10^−3^	0.979	0.080	0.024
Fold 5	1.1 × 10^−3^	0.982	0.056	0.023
Fold 6	2.1 × 10^−3^	0.965	0.114	0.030
Fold 7	1.5 × 10^−3^	0.979	0.082	0.026
Fold 8	2.7 × 10^−3^	0.958	0.070	0.034
Fold 9	1.6 × 10^−3^	0.961	0.108	0.033
Fold 10	1.3 × 10^−3^	0.978	0.085	0.027
Mean	1.6 × 10^−3^	0.973	0.080	0.027
STD	0.4 × 10^−3^	9.0 × 10^−3^	18.7 × 10^−3^	3.8 × 10^−3^

**Table 5:** Performance measures for analysis of different input variable combinations.

Combinations	Performance Measures						
	MSE	I		SI		R^2^	
		Test	Train	Test	Train	Test	Train
1: X_1_, X_2_, X_3_, X_4_, X_5_, X_6_, X_7_	0.8 × 10^−3^	0.997	0.999	0.058	0.032	0.988	0.999
2: X_2_, X_3_, X_4_, X_5_, X_6_, X_7_	1.3 × 10^−3^	0.995	0.999	0.073	0.040	0.981	0.998
3: X_1_, X_4_, X_5_, X_6_, X_7_	1.1 × 10^−3^	0.996	0.999	0.066	0.070	0.984	0.995
4. X_1_, X_2_, X_3_, X_4_, X_6_	1.6 × 10^−3^	0.994	0.999	0.082	0.041	0.977	0.998
5. X_1_, X_4_, X_5_, X_7_	1.3 × 10^−3^	0.995	0.999	0.072	0.071	0.982	0.995
6. X_2_, X_3_, X_4_, X_6_	22.5 × 10^−3^	0.894	0.967	0.303	0.351	0.675	0.845
7. X_2_, X_3_, X_6_	22.9 × 10^−3^	0.889	0.962	0.307	0.370	0.668	0.820
8. X_1_, X_5_, X_7_	3.7 × 10^−3^	0.986	0.987	0.123	0.230	0.947	0.948

**Table 6:** Statistical parameters of the results.

Methods	MSE	I	SI	R^2^
Van der Meer (2005)	9.0 × 10^−3^	0.974	0.221	0.810
D’Angremond (1996)	6.0 × 10^−3^	0.983	0.161	0.840
Bleck and Oumeraci (2001)	17.0 × 10^−3^	0.960	0.238	0.710
Gradient boosting	0.8 × 10^−3^	0.997	0.058	0.988

## Appendix A

**Table A1:** The indication of the range of the parameters.

Parameter	Definition	Average	Max	Min
H_0_ (m)	Wave height	0.123	0.231	0.021
T_0_ (s)	Wave period	2.017	3.660	0.910
R_C_ (m)	Crest freeboard	−1.081	0.196	−0.420
B (m)	Crest width	0.429	1.000	0.200
Dn_50_ (m)	Nominal diameter	0.110	0.161	0.028
tanα	Slope of structure	0.507	0.667	0.250
